# Baiting out a full length sequence from unmapped RNA-seq data

**DOI:** 10.1186/s12864-021-08146-4

**Published:** 2021-11-27

**Authors:** Dongwei Li, Qitong Huang, Lei Huang, Jikai Wen, Jing Luo, Qing Li, Yanling Peng, Yubo Zhang

**Affiliations:** 1 Animal Functional Genomics Group, Shenzhen Branch, Guangdong Laboratory for Lingnan Modern Agriculture, Genome Analysis Laboratory of the Ministry of Agriculture, Agricultural Genomics Institute at Shenzhen, Chinese Academy of Agricultural Sciences, Shenzhen, 518120 China; 2 Guangdong Provincial Key Laboratory of Protein Function and Regulation in Agricultural Organisms, College of Life Sciences, South China Agricultural University, Guangzhou, Guangdong 510642 China; 3 Animal Breeding and Genomic, Wageningen University & Research, Wageningen, 6708PB, Netherlands

**Keywords:** Unmapped reads, Full length sequence, Statistical model, RNA-seq

## Abstract

**Background:**

As a powerful tool, RNA-Seq has been widely used in various studies. Usually, unmapped RNA-seq reads have been considered as useless and been trashed or ignored.

**Results:**

We develop a strategy to mining the full length sequence by unmapped reads combining with specific reverse transcription primers design and high throughput sequencing. In this study, we salvage 36 unmapped reads from standard RNA-Seq data and randomly select one 149 bp read as a model. Specific reverse transcription primers are designed to amplify its both ends, followed by next generation sequencing. Then we design a statistical model based on power law distribution to estimate its integrality and significance. Further, we validate it by Sanger sequencing. The result shows that the full length is 1556 bp, with insertion mutations in microsatellite structure.

**Conclusion:**

We believe this method would be a useful strategy to extract the sequences information from the unmapped RNA-seq data. Further, it is an alternative way to get the full length sequence of unknown cDNA.

**Supplementary Information:**

The online version contains supplementary material available at 10.1186/s12864-021-08146-4.

## Background

RNA-seq is a pervasive tool for characterizing alternative splicing events and new RNA species [1, 2]. However, RNA-seq data is always not fully mined. In traditional RNA-seq analysis, unmapped reads are usually disregarded and discarded as futility [3, 4]. In recent years, more and more researchers start focusing on unmapped data and salvaging valuable information. Kazemian M et al. assembled novel transcripts by human unmapped RNA-seq data and found they are associated with cancer [5].

Except for sequencing errors, unmapped reads can arise due to the technical deficiencies of aligner and the existence of unknown transcripts [6]. Recently, researchers have developed different methods to cope with these demerits. Some strategies realign unmapped reads for mining valid data. For instance, RAUR (Re-align the Unmapped Reads) provides an algorithm to increase the alignment rate of low quality unmapped reads [7]. Others reassemble unmapped reads by traditional assembly tools, such as Trinity [8] and ABySS [9]. Taking Comprehensive Assembly and Functional annotation of Unmapped RNA-Seq data (CAFU) for example, this software can extract and de novo assemble unmapped reads from a single- and mixed- species samples [10]. However, current assembly tools such as Cufflinks [11] and Stringtie [12] are designed to eliminate low abundance transcripts [12]. The regions with low coverage and sparse signal might be neglected, even though they can also be valid. It is a technical challenge to reconstruct low abundance transcripts, as their reads number is too low to assemble accurately [13]. Whereas, many noncoding RNAs are expressed at low levels [14].

Comparing with random primers of standard RNA-seq, gene-specific primers can enrich interest regions average > 100 times [15]. Therefore, we design specific reverse transcription primers (SRTPs) to capture its both ends. Then, coupling next generation sequencing with a statistical model to estimate the full-length transcript, and validate it by Sanger sequencing.

## Results and discussion

**Fig. 1:**
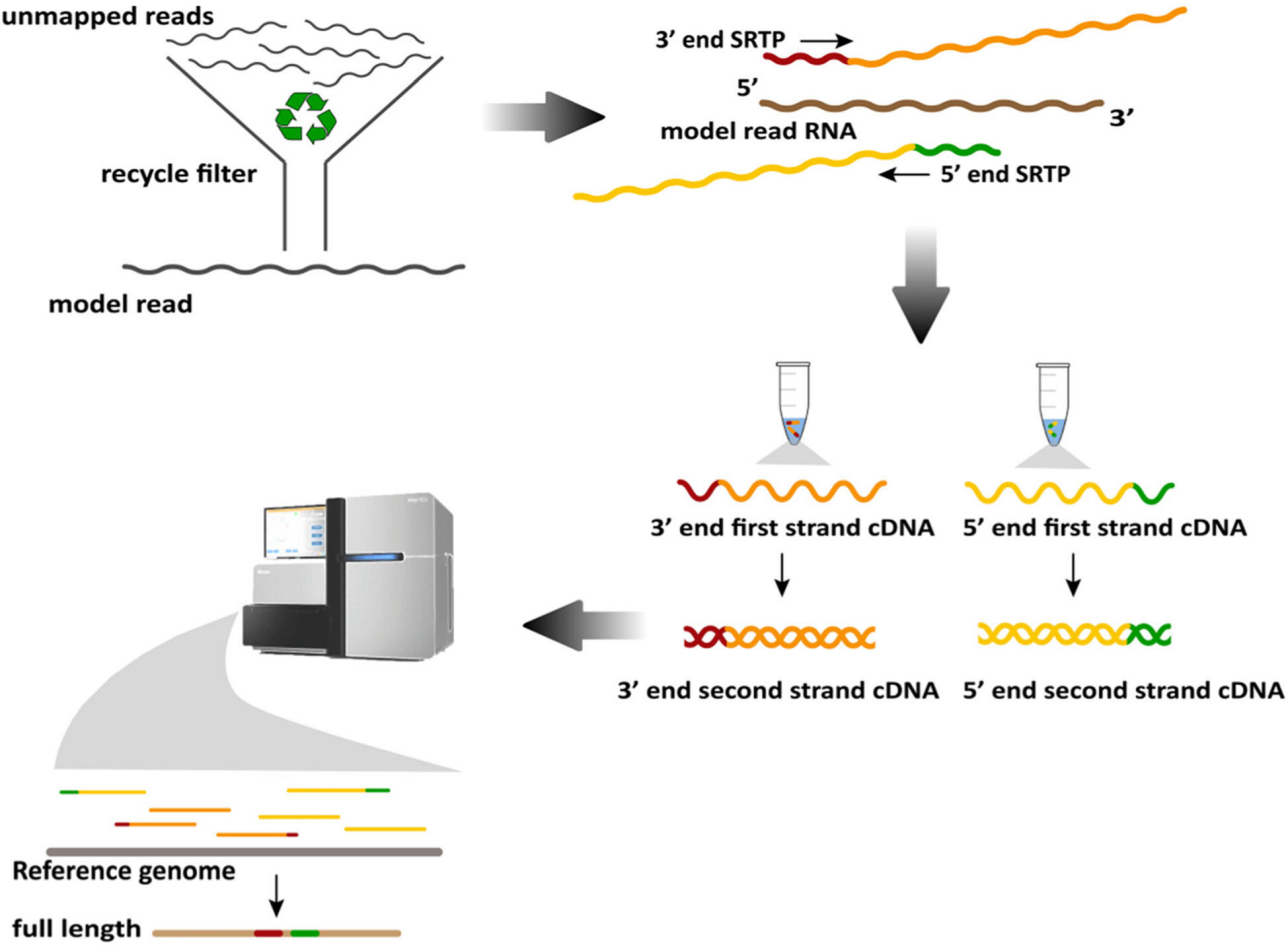
Schematic overview of this strategy. Model read is selected from the screened unmapped reads. 5′ end SRTP (5′ end specific reverse transcription primer, green single strand) and 3′ end SRTP (3′ end specific reverse transcription primer, red single strand) are designed along the sequence of model read RNA and used to synthesize the first strand cDNAs. After the second strand cDNAs are synthesized and sequenced, the reads are mapped to reference genome and used to estimate the full length sequence. The clipart of Hiseq 2500 depicted in Fig. 1. is downloaded from Illumina official site (https://www.illumina.com/systems/sequencing-platforms/hiseq-2500.html) with the authorization

The outline of this strategy procedure is depicted in Fig. 1. For recycling unmapped reads which from RNA-seq data, we carry out a rigorous screening process. After that, we randomly select one of unmapped reads as a model. To capture its both ends and increase reads enrichment, we design SRTPs to produce the first strand cDNAs. In this step, 5′ end reverse transcription can be carried out by SRTP directly. However, in conventional methods, 3′ end reverse transcription needs to introduce adapter to 3′ end first. As we consider that the direction of primer amplification depends on the direction of itself rather than template, we design 3′ end SRTP to share the same direction and complementary with RNA. In this case, we can capture 3′ end cDNAs by using 3′ end SRTP. For obtaining the full length sequence information by Illumina platform, second strand cDNAs are synthesized and combined to ultrasonic disrupted to construct library. After sequencing, we estimate the full length by a statistical model and validate by Sanger sequencing.

Unmapped reads are obtained from RNA-seq data (GSM3188619). The recycling workflow and the proportion of each part are depicted in Additional file 1: Fig. S1a-b and Additional file 2: Table S1. To filter biased reads, we classify the unmapped data into two types, including ineffective reads and new annotated fragments. Ineffective reads which account for roughly 35.8% (unpaired reads, 32.4%; mitochondrial RNA, ribosomal RNAs, bacteria and virus, 3.2%; low quality reads, 0.2%) are filtered out. To exclude the differences between different versions of the transcriptome library, we filter the reads (46.4%) that have been annotated in the mm10 version database (http://hgdownload.soe.ucsc.edu/goldenPath/mm10/bigZips/genes/mm10.refGene.gtf.gz). After eliminate low score reads (16.70%), the rest are subjected to remove new annotated transcripts (0.46%). The above screening resulted in a collection of 15,580 (0.58%) valid unmapped reads. These reads are as the candidate dataset. According to Inchworm, the reads containing a gap which size is higher than 20 bp hold higher probabilities to be introns [16]. Therefore, we pick out 36 reads with this feature from the candidate dataset.

To demonstrate the efficiency of this method, we randomly select one 149 bp candidate read as a model (Additionally file 2: Table S2). It is worth noting that there is a 23 bp gap in the locations of chr13: 64,787,091 to 64,787,115 (Additional file 1: Fig. S1d), so we infer that this gap is the reason why the model is discarded in normal alignment. To verify the model is real existence rather than sequencing error, we perform PCR followed by Sanger sequencing (see methods). In the result, the size of PCR product is 101 bp, and the 23 bp gap does exist (Additional file 1: Fig. S1c-d).

After synthesis of first and second strand cDNAs and fragmented them by ultrasonic, we obtain 9.4 ng double strand cDNA fragments and use them for library construction. When detecting on E-Gel, the library has a smear distribution and the mainly bands are concentrated at the size of approximately 320 bp. As RT (reverse transcription) products may have different sizes, so in order not to lose information of small fragments, the library between adapter dimer to 700 bp is recovered (Additional file 1: Fig. S2). Ultimately, we get a 110.88 ng library.

**Fig. 2:**
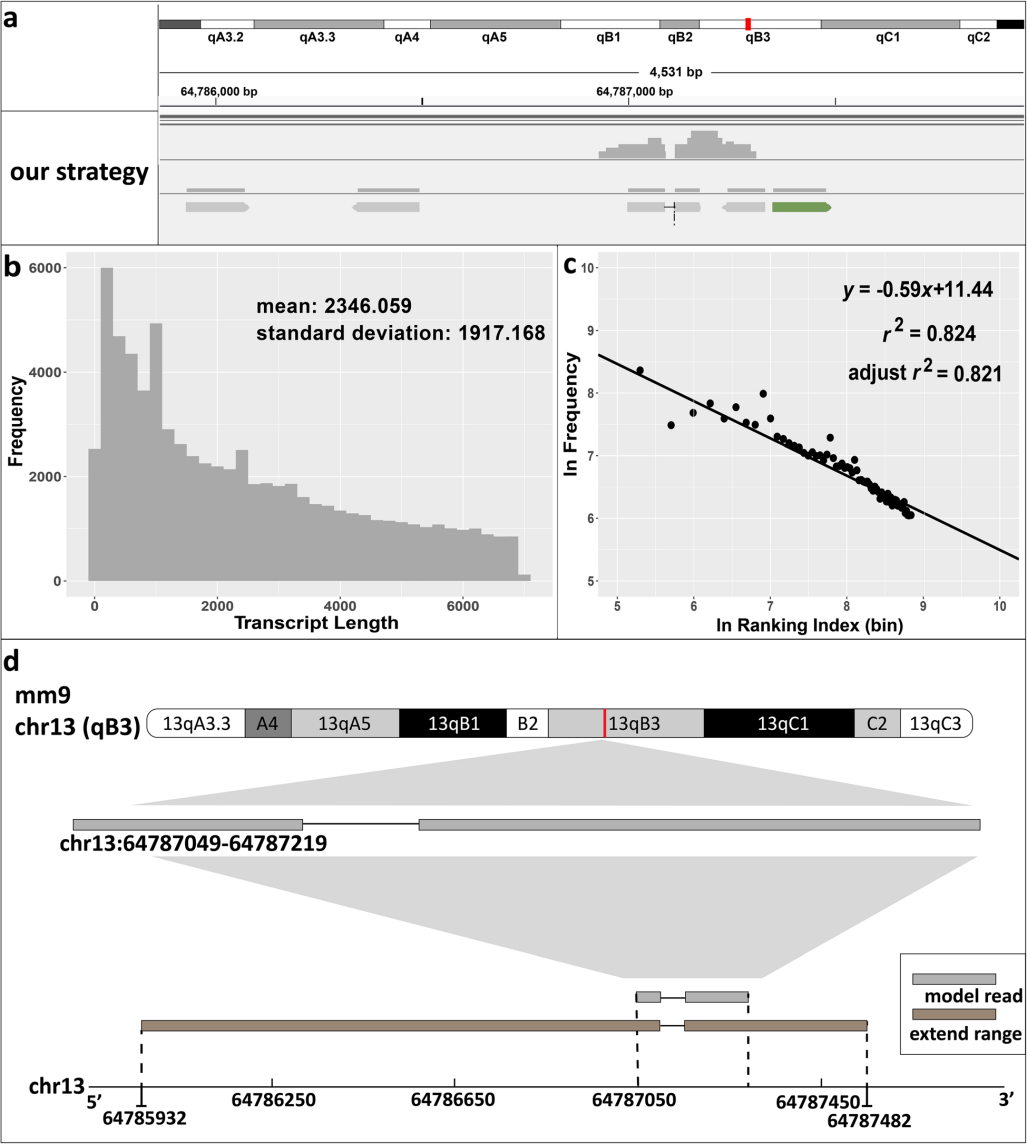
Estimation of the full length sequence. **a** Integrative Genomics Viewer (IGV) shows the distribution of the reads which nearby the model read. **b** Frequency histogram shows the length distribution of mouse transcripts. **c** Linear regression plot shows the length of mouse transcripts following power law distribution. Horizontal axis is the logarithmic value of binning ranking index, and longitudinal axis is the logarithmic value of frequency of transcript length. **d** Schematic diagram presents the sizes and the locations of model read (gray) and estimated full length sequence (brown)

Next, we estimate the full length by statistical model. Illumina sequencing shows that our model read is located in a low coverage region (Fig. 2a). It is inevitable that low sampling fraction may result in exons at low levels of enrichment [17]. Therefore, conventional assembly strategies can not be applied to reconstruct the full length of low coverage transcript. We design a statistical model to estimate the probabilities of fragments which are a part of the novel transcript. It is reported that the distribution of mouse exon lengths shows a very clean power law decay [18]. With this theory, we select potential fragments by validating the distribution of mouse transcript and calculating the cumulative distribution function.

$$ F(x)=1-\frac{C}{a-1}{x}^{-\left(a-1\right)} $$

Where x is the length which from the model center to the candidate fragment center. C is the normalization constant. Alpha is the unstandardized coefficients of the power low distribution.

Firstly, we verify that the mouse transcripts’ lengths also follow power law distribution (Fig. 2b, c). Moreover, researchers find that longer transcripts produce more reads relative to shorter transcripts of similar expression levels in high-throughput sequencing [19]. To lessen the bias of this technical feature, we filter the fragments of which the lengths are higher than median (6932 bp) to gather the uniform data set. We use log-log method to fit the parameters and set 100 as the bin size. We get the adjusted R square is roughly 0.821 by linear regression (Fig. 2c), and the unstandardized coefficient is 0.594 with the significant level lower than 1e10–3. It indicates that the lengths of mouse transcripts follow power law distribution with a high significance. Then we get the parameter alpha (0.594) and normalization constant (92,637.295). The fragments have probabilities higher than 0.3 are considered to be in the same transcript. Taking the model site (chr13 64,787,049) as the center, we calculate the probabilities of fragments in the adjacent range. Finally, we gain 5 reads for downstream verification (Fig. 2a).

In total, the full-length estimated by statistical model is 1530 bp. There are 263 bp extensions from 3′ end and 1117 bp extensions from 5′ end, has a total 1380 bp extensions (Fig. 2d). Coincidentally, 3′ end extension including 54 bp continuous GT repeats followed by 58 bp continuous GA repeats (Additional file 2: Table S2). It is a typical microsatellite structure which is difficult to sequence. It has been reported that GT repeats in promoter is associated with diseases [20].

Next, to validate the estimated full length sequence obtained by statistical model, we perform RT-PCR using specific primers followed by Sanger sequencing. It is worth noting that when align to mouse mm9 assembly, there are 10 bp TG deletions in the locations of chr13: 64,787,269 to 64,787,280. Meanwhile, there are 34 bp insertions in the locations of chr13: 64,787,329 to 64,787,330 (Additional file 1: Fig. S3a, b). These mutations occur in the microsatellite structure. This discrepancy between NGS platform and Sanger sequencing might be attributed to lower coverage and depth in microsatellite structures [21]. In total, the full length of model is 1556 bp, and has 1407 bp extend (Additional file 2: Table S2).

## Conclusions

Unmapped reads have been proved useful but are always discarded in RNA-seq. Our recycle process can extract valid unmapped reads. Combining SRTP amplification with statistical model, we obtain its full length sequence. In summary, our strategy provides a useful tool to mine the information which holds in unmapped reads. This protocol can be applied in determining the full length when model reads are located in low abundance. In addition, this strategy can be used as an alternative to traditional RACE [22] to amplify the full length sequence of unknown cDNA. Simultaneously, it is low cost and easy to operate.

## Methods

### Extraction of unmapped reads and selection of model read

Unmapped reads were obtained from RNA-seq data (data processing see Additional file 3), and then subjected to bam2fastq directly to transfer data format and remove unpaired reads. The remaining reads were screened by SortMeRNA [23] to remove mitochondrial, ribosomal RNAs, bacteria and virus sequences as well as other non mouse sequences with the current version database. Fastp [24] was used to filter out low quality reads with default arguments. The resulting unmapped reads were then aligned to GTF of mm10 version by HISAT2 [13] and TopHat [25]. Then we filtered the union of mapped reads. Following the above steps, BLAT [26] was used to extract high score reads (minscore = 130). After BLAST [27] to reference genome, new annotated transcripts were excluded. We then screened the reads with a gap which is higher than 20 bp as candidate reads and randomly selected one of them as a model for the following step.

### Cell culture

Mouse embryo stem cells were cultured according to *Jérome Chal*’s method [28]. In detail, mESs cells which removed feeders were grown at 37 °C in a 5% CO2 incubator in DMEM supplemented with 15% fetal bovine serum (Gibco; 10099–141), Penicillin-Streptomycin, 0.1 mM nonessential amino acids (NEAA; 11140–050), 0.1 mM β-mercaptoethanol (Gibco; 21985–023), 1% Sodium pyruvate (Gibco; 11360–070), 1000 U/ml LIF (Millipore; ESG1107) and 2i inhibitors, on gelatin-coated (0.1% v/v) plates. The culture medium was changed daily. After three days of culture, the cells were lysed directly using TRIzol (Invitrogen; 15596026) for 5 min, and then directly to isolate RNA or store at − 80 °C.

### RNA isolation

Total RNA was isolated using TRIzol reagent according to the manufacturer’s protocol. The quality of the total RNA was measured using a Qubit® 3.0 Fluorometer (Life Technologies; USA). The quality of the total RNA was evaluated using Thermo Scientific NanoDrop 2000c spectrophotometer and agarose gel electrophoresis. RNA had 260/280 ratios > 1.8 and 260/230 > 2.0.

### Model read identified by sanger sequencing

The PCR primers which flank the gap of model read were generated using Primer3, and their specificity was tested by UCSC In-Silico PCR (Additionally file 2: Table S2). RT was performed with random hexamers of SuperScript III First-strand Synthesis System for RT-PCR (Invitrogen) according to the manufacturer’s instructions. PCR was carried out with PrimeSTAR Max DNA Polymerase (TaKaRa; R045A) in 25 μl volume. The reaction conditions were as follows: initial denaturation at 98 °C for 5 min; 35 cycles of 98 °C for 10 s, 60 °C for 15 s, 72 °C for 20 s; and a final extension at 72 °C for 10 min. The PCR products were run on polyacrylamide gel electrophoresis (PAGE) and the band at approximately 101 bp was separated for gel recovery.

Gel recovery was conducted as follows: Three repeated PCR reactions were performed respectively. Gel slices were placed into 0.5 ml microcentrifuge tube which pierced with 21 G needle, and then put into 1.5 ml RNase-free microcentrifuge tube. Centrifuged at 20,000 g for 4 min at room temperature. 200 μl of RNase-free H_2_O was added and mixed. The mixtures were then incubated at 70 °C for 10 min and vortex for 30 s at a medium intensity setting. The gel slurry was transferred into a microcentrifuge tube filter and centrifuged at 20,000 g for 3 min at room temperature. The eluates of three repeated PCR reactions were combined and added 25 μl of 5 M NaCl. The DNA was precipitated by adding 12 μl 20 μg/μl Glycogen (Invitrogen; 10814–010) and 750 μl of isopropanol, then incubated at − 20 °C overnight. The DNA was pelleted by centrifuging at 20,000 g for 30 min at 4 °C. Then, the DNA pellet was washed by 750 μl of 80% (vol/vol) ice-cold ethanol and centrifuge at 20,000 g for 2 min at 4 °C. Finally, the DNA pellet was placed at room temperature to air-dry for 10 min and resuspended in 15 μl H_2_O.

The recovery products were ligated to pClone007 Blunt Simple vector (TSINGKE; TSV-007BS-a) and then transformed to Trelief™ 5α competent cells (TSINGKE; TSC01). The result of Sanger sequencing was aligned with model read using MegaX software.

### Specific reverse transcription primers design

SRTPs were designed according to the principle described above. At the same time, they should meet the general requirements for primers itself and can exclude off-target amplification. The location of SRTPs is relatively not strictly restricted, but the products of both ends should have overlap in case of losing sequence information. In this study, we directly used forward PCR primer as 5′ end SRTP and the complementary sequence of reverse PCR primer as 3′ end SRTP (Additionally file 2: Table S2).

### The first strand cDNA synthesis

5′ and 3′ end first strand cDNAs were prepared separately. In order to meet the starting material requirement of library construction, 1 μg total RNAs are used for each reaction. Ribosomal RNA was removed from total RNA using Ribo-off rRNA Depletion Kit (Vazyme; N406–01). The ribosomal-depleted RNA was purified by using 2.2X volume RNA beads (Vazyme; N412–01) and eluted in 10 μl of Nuclease-free H_2_O. We synthesized the cDNA using SuperScript® III First-Strand Synthesis System for RT-PCR (Invitrogen; 18080–051). In detail, 1 μl of 2 μM specific reverse transcription primer and 1 μl of 10 mM dNTP mix were added to 8 μl of RNA sample. The mixture was incubated at 65 °C for 5 min and the place on ice > = 1 min. An equivalent volume of cDNA synthesis mixture which contains 10X RT buffer, 25 mM MgCl_2_, 0.1 M DTT, RNaseOUT™ (40 U/μl), and SuperScript® III RT (200 U/μl) was added to the RNA/primer mixture, resulting in a total of 20 μl reaction volume. The mixture was incubated at 50 °C for 50 min and terminated at 85 °C for 5 min. Next, the first strand cDNA mixture was treated with 1 μl of RNase H for 20 min at 37 °C. Then, the mixture was purified with 1.2X volume of DNA beads (Vazyme; N411–01) and eluted in 42.5 μl of Nuclease-free H_2_O.

### The second strand cDNA synthesis and ultrasonic fragment

5′ and 3′ end second strand cDNAs were synthesized respectively. For the second strand cDNA synthesis, 5 μl of 10X second strand buffer and 1.5 μl of 10 mM dNTP mix were added to 40.5 μl of purified first strand cDNA and then incubated on ice for 5 min. Next, 2.5 μl of DNA polymerase I, E.coli (10 U/μl) (NEB; M0209L) and 0.5 μl of RNaseH (2 U/μl) was added to above first strand cDNA mixture and incubated at 15 °C for 2.5 h. 10X second strand buffer is made of 500 mM Tris-HCl (pH 7.8), 50 mM MgCl_2_ and 10 mM DTT. The buffer is filtered without DTT and then added 0.1 M DTT from Invitrogen to make final components. Second strand cDNAs were treated with 5 μl of RNase A (Thermo Scientific; EN0531) for 1 h at 37 °C and then purified by using 1.2X volume DNA beads. Finally, they were eluted in 53 μl of TE buffer.

The purified 5′ and 3′ end second strand cDNAs were combined and mixed, and then fragmented into average size of approximately 200 bp using Bioruptor Pico ultrasonic (Diagenode; USA). We used sonication conditions of 30 s on, 30 s off for 13 cycles. The products were purified by using 1.8X volume DNA beads and eluted in 53 μl of Nuclease-free H_2_O. To ensure library abundance, all of the cDNA fragments were used for library construction. The quantity was determined by Qubit.

### Library preparation and sequencing

We used VAHTSTM Universal DNA Library Prep Kit for Illumina® V3 (Vazyme; ND607) to construct library with a slightly modified manufacturer’s protocol. All of the 50 μl of purified fragment products were to perform end repair by adding 15 μl of End Prep Mix 4. Samples were incubated at 20 °C for 15 min and then 65 °C for 15 min. For adapter ligation, we added Rapid Ligation buffer 2, Rapid DNA ligase and adapter to end repair products and incubated at 20 °C for 30 min. After 1.2X volume DNA beads purified, the adapter ligation products were eluted in 10 μl of Nuclease-free H_2_O. For library amplification, 2 μl of PCR Primer Mix 3 for Illumina and 10 μl of VAHTS HiFi Amplification Mix were added to 8 μl of adapter ligation products. PCR cycling conditions consisted of initial denaturation at 95 °C for 3 min; 7 cycles of 98 °C for 20 s, 60 °C for 15 s, 72 °C for 30 s; and a final extension at 72 °C for 5 min. Library was run on 2% E-Gel (Invitrogen). The fragments which size between adapter dimer to 700 bp were recovered using Zymoclean Gel DNA Recovery Kit (Zymo Research, D4008). The library was sequenced on Illumina Novaseq platform, which generated 150 bp paired-end reads.

### Estimated full-length sequence of model read

#### NGS data processing

NGS reads were trimmed by cutadapt (1.8.dev0) [29] and mapped with bowtie2 (version 2.1.0) [30] to the mm9 with local alignment strategy. The fragments which aligned in chr13 were kept for downstream analyses.

#### Verify mouse transcripts length distribution

To verify the length distribution of mouse transcripts, we downloaded the gene transfer format file (gencode.v25.annotation.gtf.gz) from Gencode. Then we extracted the length information, filter overlong transcripts and set 100 as bin size to compute the frequency. The length higher than sample median was considered as overlong transcripts. We used the logarithmic frequency and bin index to perform a linear regression to verify if the data obeys a power-law distribution.

#### Power-law parameters and transcript extension estimation

Alpha value is computed by ordinary least squares with ln x (ranking index) and ln y (frequency). The parameters C are calculated by IBM SPSS Statistics 19 (IBM SPSS) programme. Then, we classify the fragments adjacent to the model reads based on the distance, and calculate the probability. Finally, fragments with probability greater than 0.3 are retained for downstream validation.

#### Validated its full length sequence

Specific primers were designed at both ends of sequence estimate by statistical model and each primer has a length of 21 bp (Additionally file 2: Table S2). Reverse transcription was performed using Superscript III system and random primer. PCR was carried out with Phusion High-Fidelity DNA Polymerase (Thermo Scientific; F530S). The reaction condition was as follows: Initial denaturation at 98 °C for 30 s; 35 cycles of 98 °C for 10 s, 55 °C for 30 s, 72 °C for 45 s; and a final extension at 72 °C for 10 min. The PCR products were detected on agarose gel electrophoresis and the target band was sliced and recovered by Zymoclean Gel DNA Recovery Kit. The purified products were ligated to pCE2 TA/Blunt-zero vector (Vazyme; C601–02) and transformed to Trelief™ 5α competent cells. Single clones were picked and subjected to Sanger sequencing, and the result sequence was aligned to mouse reference genome by BLAT of UCSC genome browser [31].

## Supplementary Information

**Additional file 1: **Supplementary figures. **Fig. S1.** Screening valid unmapped reads and verifying the model read. **Fig. S2.** Library recovered by E-Gel. **Fig. S3.** Validate the full length sequence by Sanger sequencing.

**Additional file 2: **Supplementary tables. **Table S1.** Process and results of valid unmapped reads recycle. **Table S2.** Sequences of Model read and its full length and primers used in this study.

**Additional file 3:.** RNA-Seq data processing. The RNA data was sequenced (paired-end 150 base pairs [bp]) using the Illumina platform. Trimmomatic (version 0.36) [1] were applied to clean the adapter-containing reads, poly-N-containing reads, and low-quality reads. Clean data was aligned to the NCBI37/mm9 reference genome using TopHat v2.0.12 [2] with the parameters --read-mismatches and --library-type were set to 5 and fr-firststrand.

## Data Availability

RNA-seq data can be obtained at the NCBI Gene Expression Omnibus (https://www.ncbi.nlm.nih.gov/geo) under the accession number GSM3188619. All deep sequencing data generated in this paper have been submitted to the NCBI Gene Expression Omnibus (https://www.ncbi.nlm.nih.gov/geo) under the accession number GSE172487 [32]. The methods in detail of this study are included within the article and its additional files. The computational analysis pipeline of unmapped reads recycling is included in additional file 2. Biological materials used in this study available from the corresponding author.

## Abbreviations

bp: base pair
CAFU: Comprehensive assembly and functional annotation of unmapped RNA-Seq data
GTF: Gene transfer format
IGV: Integrative genomics viewer
NGS: Next-generation sequencing
PCR: Polymerase chain reaction
PAGE: Polyacrylamide gel electrophoresis
RAUR: Re-align the unmapped reads
RT: Reverse transcription
RT-PCR: Reverse transcription-polymerase chain reaction
SRTP: Specific reverse transcription primer
UCSC: University of California Santa Cruz
