# Supplementary material for: Baiting out a full length sequence from unmapped RNA-seq data
Source: BMC Genomics. 2021 Nov 27;22:857. doi: 10.1186/s12864-021-08146-4 (PMC8626966; doi:10.1186/s12864-021-08146-4)
Supplement: Supplementary file 1 — Additional file 1: Supplementary figures. Fig. S1. Screening valid unmapped reads and verifying the model read. Fig. S2. Library recovered by E-Gel. Fig. S3. Validate the full length sequence by Sanger sequencing. [file 12864_2021_8146_MOESM1_ESM.docx]

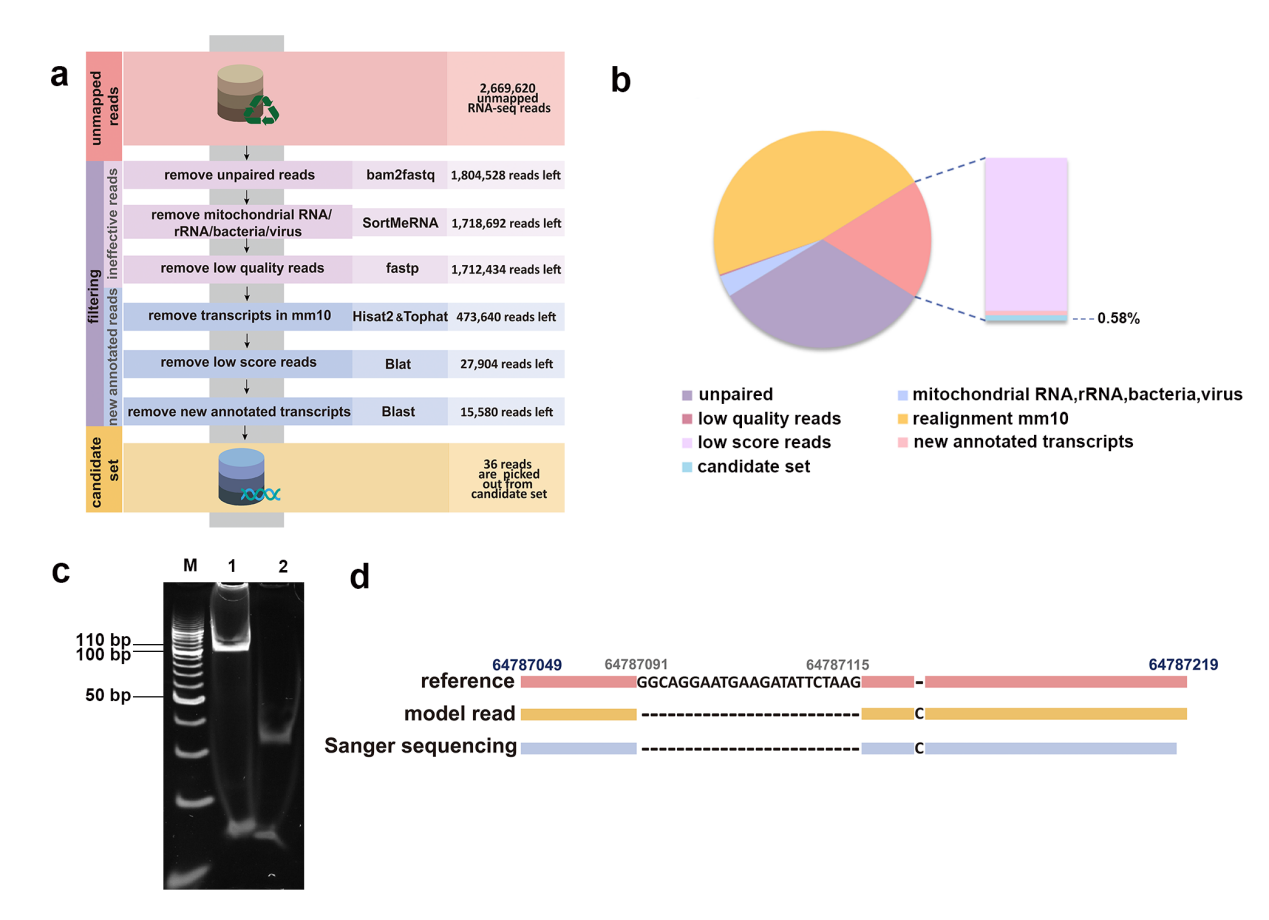


**Figure S1.** Screening valid unmapped reads and verifing the model read. **a** Data processing strategy and softwares for screening valid unmapped reads. **b** The pie chart illustrates the proportion of reads after screening as described in **a**. **c** Detecting PCR amplification of model read by PAGE. M: 10 bp DNA ladder; 1: PCR products of model read; 2: Negative control. **d** Identification of model read by Sanger sequencing. Dotted lines represent a 23 bp gap identified by both model read and Sanger sequencing.


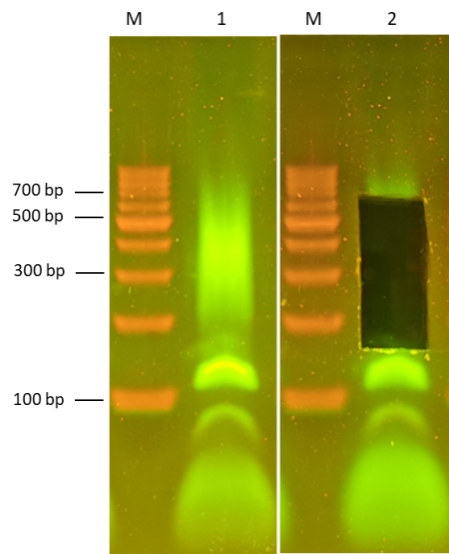


**Figure S2.** Library recovered by E-Gel. M: 100 bp DNA ladder; 1: Library running on E-Gel; 2: Recovering the library from adapter dimer to 700 bp.


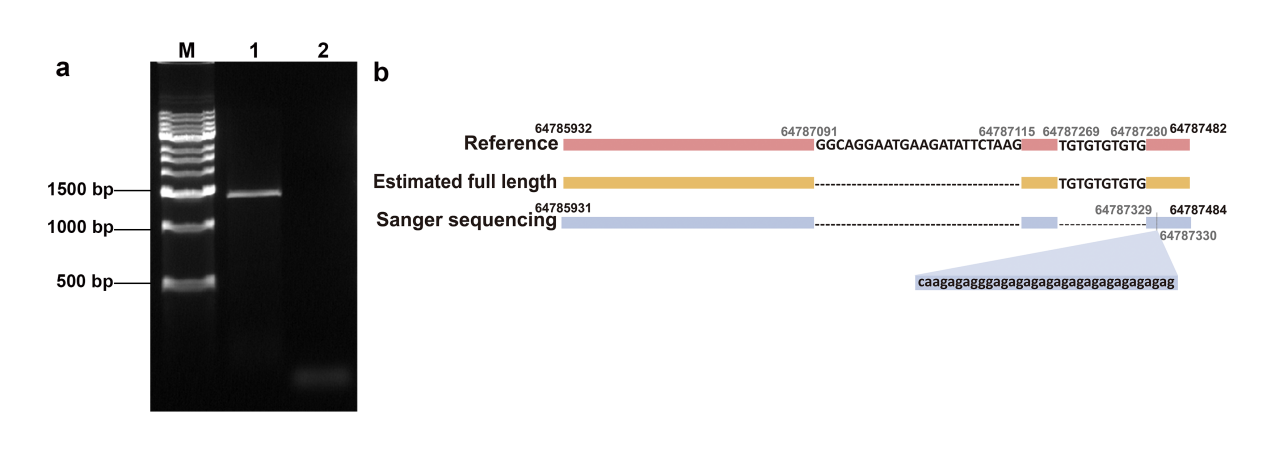


**Figure S3.** Validating the full length sequence by Sanger sequencing. **a** Detecting PCR amplification of full length sequence by agarose gel electrophoresis. M: 1 kb DNA ladder; 1: PCR products of the full length sequence; 2: Negative control. **b** Identification of the full length sequence by Sanger sequencing. The long dotted lines represent the 23 bp gap identified by both estimated full length and Sanger sequencing. The short dotted line represents the 10 bp TG deletions. Additionally, there are 34 bp insertions in Sanger sequencing.
