# Supplementary material for: Baiting out a full length sequence from unmapped RNA-seq data
Source: BMC Genomics. 2021 Nov 27;22:857. doi: 10.1186/s12864-021-08146-4 (PMC8626966; doi:10.1186/s12864-021-08146-4)
Supplement: Supplementary file 2 — Additional file 2: Supplementary tables. Table S1. Process and results of valid unmapped reads recycle. Table S2. Sequences of Model read and its full length and primers used in this study. [file 12864_2021_8146_MOESM2_ESM.docx]

Table replacement instructions:

We found a few typos when we checked the manuscript thoroughly. Table S1 has been updated. This does not affect the results. Accordingly, we have corrected the percentage of low score reads from 16.68% to 16.70% in main text on the online correction.

**Table S1.** Process and results of valid unmapped reads recycle.

Old version Table S1.

| **Filtered** | **Num** | **Rest** | **Tool** |
| --- | --- | --- | --- |
| total | 2669620 | 2669620 |  |
| unpaired | 865092 | 1804528 | bam2fastq |
| mitochondrial RNA | 85836 | 1718692 | sortmerna |
| low quality reads | 6256 | 1712434 | fastp |
| realignment mm10 | 1238794 | 780534 | tophat/bam2fastq |
|  |  | 476484 | Hisat2 |
|  |  | 473640 | bam2fastq |
| low score reads & unaligned | 445376 | 515112 | bowtie2/samtools |
|  |  | 49728 | bam2fastq |
|  |  | 59652 | blat |
| new annotated transcripts | 12324 | 15580 | blast |

Updated version Table S1:

| **Filtered** | **Num** | **Rest** | **Tool** |
| --- | --- | --- | --- |
| total | NA | 2,669,620 |  |
| unpaired | 865,092 | 1,804,528 | bam2fastq |
| mitochondrial RNA | 85,836 | 1,718,692 | SortMeRNA |
| low quality reads | 6,258 | 1,712,434 | fastp |
| realignment mm10 | 1,238,794 | 780,534 | Tophat/bam2fastq |
|  |  | 476,484 | Hisat2 |
|  |  | 473,640 | bam2fastq |
| low score reads & unaligned | 445,736 | 62,775 | bowtie2/samtools |
|  |  | 49,728 | bam2fastq |
|  |  | 27,904 | Blat |
| new annotated transcripts | 12,324 | 15,580 | Blast |

**Table S2.** Sequences of Model read and its full length and primers used in this study.

| Name |  |  |
| --- | --- | --- |
| PCR primers | Model read F | CTGGTGCCATAATTCAGGGA |
|  | Model read R | CCTAGAAGTGCATTGCCTAACA |
|  | Full length F | TTCTGAAGACTCTACCTAAAG |
|  | Full length R | ATAAATAAAGTACAAGAGGTA |
| Specific reverse transcription primers | 5' end SRTP | CTGGTGCCATAATTCAGGGA |
|  | 3' end SRTP | GGATCTTCACGTAACGGATTGT |
| Model read sequence | CTGGTGCCATAATTCAGGGAACTGTGTTCTTGATGTACTATCTGAGACATTTGTGCTTCCCCCCATCCAGCTATCAGGCTGTTAGGCAATGCACTTCTAGGAATTAGAATTCTATAAGGAATCTCATGCTGGAAGAACAAAAAGACCCA | |
|  |  |  |
| Full length sequence estimated by statistical model | TTCTGAAGACTCTACCTAAAGGAGCTGATAGATAAAACCATTGTAAAGTTAATGAGTTCAGGGACCAGAGAAGTAACAGAAACGATAAAAATTATCTAGGGTAGAAGTCAACACTAAATAAAAAGAAACCAAATCAAGGCCCCGATGCCTCCTCTAGGGAGACTTTGATGCCGGCTTGTCAAAGTTAGACTTTTTTGTTGTTAGCAGTTGAAAGTGTAGATCTCTTTTTCATTGGTGTCTAGGTTCACAGCTGTGGTTTGTCATGAGGGGGACTTGGCTTTTGCATCAAATGGTTTCAACTGGTTTGACACACTTTCATGTTCCAGACAACACCTCACCCTTTGTTATTTTGTTATTAGCCAGAGGCTCCATTACTATAATGAATACCTGAGAGAAATTGTCTAGAAAGAAAACCTCATTTTGGCTCACAGCTTCAGAGATTTTGGTTTATGATCAGCTATTTCTGTTACTTTTACAACTATAGTAAGGTAGGCACATCTTGGTGGAAGGACATGGTGGCAGAAAATTTCTCACTTCTGGAAGCCAGGGAACAGGAATAAATCAGGAAGAAGAAAAGGGCAAGATGTACTTCTTAAGGCACATACCCCATGTGATCCATTCCCACCACATAGGCCTCAATTCCAGAGTTCTACCACATCACAGTAGTCTCCTTGAAATTTGATACTGCTGGTGAATAAGACAGTGAATTAGTCAGCATCATGTTGATATGTCTCTTAAGTGCCACCAGAGACATACCCAGAGGTGTGCTTTATTTGTATTGAGATGTTTCCAACCATTCAAGATTAACCACCACAGTTACCATCTGACTCATGTCTCATGATTGTCTGCAGCAAAGCTTAGCTAGACAGCCTATGGGATTGGTGAGTCTCTTCTGACTTTTCTTGGGACAGATGAATAAACATCTGTCCATTCTAGTATTGGTACAGATCAAAGAAACAATCACACCCAATTCTATTTTGGTAAGCCAGTGACTTTATTGGGGTTACTTACAGGAGTGTGGATGACGCAAAGGTGGATGTACCACTGAAAAGCCCACCCCAGCATGGTGATGACTCATGAAAGCGGAATCCCTGGCATACTCTCTGTTCCTAAATTTCTGGTGCCATAATTCAGGGAGACTGTGTTCTTGATGTACTATCTGAGACATTTGTGCTTCCCCCCATCCAGCTATCAGGCTGTTAGGCAATGCACTTCTAGGAATTAGAATTCTATAAGGAATCTCATGCTGAAAGAACAAAAAGACCCAGGCTCTGCAGTGTGTGTGTGTGTGTGTGTGTGTGTGTGTGTGTGTGTGTGTGTGTGTGTGTGTGAGAGAGAGAGAGAGAGAGAGAGAGAGAGAGAGAGAGAGAGAGAGAGAGAGAGAGAGATTATTTCTCAAACAAGATTTAATACAGTGACAACAACTTGTCAGACCATCTTGCAAGAGAGAGAGTGACTAGAATGGAGATAATAGTGACTGGACCAGACTTTGGCCGTTCTGCTTAGTCATACCTCTTGTACTTTATTTAT | |
|  |  |  |
|  |  |  |
|  |  |  |
|  |  |  |
|  |  |  |
|  |  |  |
|  |  |  |
|  |  |  |
|  |  |  |
|  |  |  |
|  |  |  |
|  |  |  |
|  |  |  |
|  |  |  |
|  |  |  |
|  |  |  |
|  |  |  |
|  |  |  |
|  |  |  |
|  |  |  |
| Full length sequence validated by Sanger sequence | TTTCTGAAGACTCTACCTAAAGGAGCTGATAGATAAAaCCATTGTAAAGTTAATGAGTtCAGGGACCAGAGAAGTAACAGAAACGATAAAAATTATCTAGGGTAGAAGTCAACACTAAATAAAAAGAAACCAAATCAAGGCCCCGATGCCTCCTCTAGGGAGACTTTGATGCCtGCTTGTCAAAGTTAGACTTTTTTGTTGTTAGCAGTTGAAAGTGTAGATCTCTTTTTCAcTGGTGTCTAGGTTCACAGCTGTGGTTTGTCATGAGGGGGACTTGGCTTTTGCATCAAATGGTTTCAACTGGTTTGACACACTTTCATGTTCCAGACAACACCTCACCCTTTGTTATTTTGTTATTAGCCAGAGGCTCCATTACTATAATGAATACCTGAGAGAAATTGTCTAGAAAGAAAgCCTCATTTTGGCTCACAGCTTCAGAGATTTTGGTTTATGATCAGCTATTTCTGTTACTTTTACAACTATAGTAAGGTAGGCACATCTTGGTGGAAGGACATGGTGGCAGAAAATTTCTcAtCTCTGGAAGCCAGGgAACAGGAATAAATCAGGAAGAAGAAAAGGGCAAGATGTACTTCTTAAGGCACATACCCCATGTGATCCATTCCCACCACATAGGCCTCAATTCCAGAGTTCcACCACATCACAGTAGTCTCCTTGAAATaTGATACTGCTaGTGAATAAGACAGTGAATTAGTCAGCATCATGTTGATATGTCTCTTAAGTGCCACCAGAGACAcACCCAGAGGTGTGCTTTATTTGTATTGAGATGTTTCCAACCATTCAAGATTAACCACCACAGTTACCATCTGACTCgTGTCTCATGATTGTCTGCAGCAAAGCTTAGCTAGACAGCCTATGGGATTGGTGAGTCTCTTCTGACTTTTCTTGGGACAGATGAATAAACATCTGTCCATTCTAGTATTGGTACAGATCAAAGAAACAATCACACCCAATTCTAtTTTGGTAAGCCAGTGACTTTATTGGGGTTACTtACAGGAGTGTGGATGACGCAAAGGTGGATGTACCACTGAAAAGCCcACCCCAGCATGGTGATGACTCATGAAAGCGGAATCCCTGGCATACTCTCTGTTCCTAAATTTCTGGTGCCATAATTCAGGGAACTGTGTTCTTGATGTACTATCTGAGACATTTGTGCTTCCCCCcATCCAGCTAtCAGGCTGTTAGGCAATGCACTTCTAGGAATTAGAATTCTATAAGGAATCTCATGCcGgAAGAACAAAAAGACCCAGGCTCTGCAGTGTGTGTGTGTGTGTGTGTGTGTGTGTGTGTGTGTGTGTaTGTGAGAGAGAGAGAGAGAGAGAGAGAGAGAGAGAGAGAGAGAGAaAGAGcaagagagggagagagagagagagagagagagagAGAGAGAGAGaTTATTTCTCAAACAAGATTTAATACAGTGACAACAACTTGTCAGACCATCTTGCAAGAGAGAGAGTGACTAGAATGGAGATAATAgTGACTGGACCAGACTTTGGCCGTTCTGCTTAGTCATACCTCTTGTACTTTATTTATAA | |
|  |  |  |
|  |  |  |
|  |  |  |
|  |  |  |
|  |  |  |
|  |  |  |
|  |  |  |
|  |  |  |
|  |  |  |
|  |  |  |
|  |  |  |
|  |  |  |
|  |  |  |
|  |  |  |
|  |  |  |
|  |  |  |
|  |  |  |
|  |  |  |
|  |  |  |
