# Supplementary material for: Baiting out a full length sequence from unmapped RNA-seq data
Source: BMC Genomics. 2021 Nov 27;22:857. doi: 10.1186/s12864-021-08146-4 (PMC8626966; doi:10.1186/s12864-021-08146-4)
Supplement: Supplementary file 3 — Additional file 3:. RNA-Seq data processing. The RNA data was sequenced (paired-end 150 base pairs [bp]) using the Illumina platform. Trimmomatic (version 0.36) [1] were applied to clean the adapter-containing reads, poly-N-containing reads, and low-quality reads. Clean data was aligned to the NCBI37/mm9 reference genome using TopHat v2.0.12 [2] with the parameters --read-mismatches and --library-type were set to 5 and fr-firststrand. [file 12864_2021_8146_MOESM3_ESM.docx]

**Supplementary file 1**: RNA-Seq data processing

The RNA data was sequenced (paired-end 150 base pairs [bp]) using the Illumina platform. Trimmomatic (version 0.36)[1] were applied to clean the adapter-containing reads, poly-N-containing reads, and low-quality reads. Clean data was aligned to the NCBI37/mm9 reference genome using TopHat v2.0.12[2] with the parameters --read-mismatches and --library-type were set to 5 and fr-firststrand.

**References for supplementary file 1**

[1] Bolger AM, Lohse M, Usadel, B. Trimmomatic: A flexible trimmer for Illumina Sequence Data. Bioinformatics.2014; 30(15):2114-2120.

[2] Trapnell C, Pachter L, Salzberg SL.TopHat: discovering splice junctions with RNA-Seq. Bioinformatics.2009; 25(9):1105-1111.
